# Ceramide Complex Ameliorates Metabolically Driven Neutrophil Senescence by Regulating Apoptosis via the cGAS-STING Pathway

**DOI:** 10.7150/ijms.104801

**Published:** 2025-02-10

**Authors:** Xi Gao, Cheng Lu, Kaixuan Wang, Chunfang Zheng, Linbin Li, Xin Zhang, Bingwei Sun

**Affiliations:** 1 Research Center for Neutrophil Engineering Technology, Affiliated Suzhou Hospital of Nanjing Medical University, Suzhou 215002, Jiangsu Province, China.; 2 Department of Critical Care Medicine, The First Affiliated Hospital of Dalian Medical University, Dalian 116000, Liaoning Province, China.

**Keywords:** Neutrophil, Senescence, Metabolically driven, Apoptosis, Ceramide, cGAS-STING pathway

## Abstract

**Background:** Population aging is increasingly recognized as a major global challenge. Researchers have identified a correlation between aging and immunosenescence, leading to dysfunction of the immune system. As a crucial component of the innate immune system, age-related changes in neutrophils have garnered significant attention from researchers, but the underlying mechanisms remain unclear. This study aims to comprehensively evaluate the senescence status and potential mechanisms of neutrophils, and to identify targets for delaying or even reversing senescence.

**Methods:** Blood routine tests and Luminex Multiplex Cytokine Analysis were employed to assess inflammation levels in mice. Flow cytometry and an agarose chemotaxis model were used to evaluate baseline biological functions and stress responses of neutrophils. Transmission electron microscopy and flow cytometry were utilized to compare mitochondrial ultrastructure and function. Metabolomic analysis was performed to examine metabolic patterns. qPCR, Western blotting, and flow cytometry were used to investigate the potential mechanisms of ceramide intervention on neutrophils.

**Results:** Our findings indicate that aged mice exhibit considerable variability in delayed apoptosis among bone marrow neutrophils, alongside a notable reduction in baseline functionality and stress response capabilities. Metabolomic analysis revealed a marked decrease in ceramide levels within aged neutrophils. *In vitro* ceramide intervention revitalized neutrophil functionality and partially inhibited delayed apoptosis, facilitating the efficient elimination of senescent neutrophils. The underlying mechanism behind these effects might be attributed to ceramide's modulation of mitochondrial permeability, which in turn influences the activation of the cGAS-STING pathway, as well as its regulatory role in maintaining the equilibrium of pro-apoptotic Bcl-2 protein levels.

**Conclusions:** This investigation proficiently assessed neutrophil senescence in terms of both biological functionalities and intrinsic diversity, while concurrently exploring the feasibility and primary mechanisms through which ceramide intervention impacts neutrophil senescence at the levels of signaling pathways, protein expression, and cellular microarchitecture. These findings provide novel insights into evaluating and potentially intervening in immune senescence, with implications for organismal aging.

## Introduction

Population aging has emerged as a pervasive global phenomenon. By the year 2030, an estimated 1.4 billion individuals worldwide will have reached the age of 60 or above, a figure that is projected to escalate dramatically to 2.1 billion by 2050, as reported by the World Health Organization. This demographic shift carries with it a significant burden of disease, posing a crucial societal challenge. Of particular note is the decline in the functionality of the immune system, a condition referred to as immunosenescence, which is a defining feature of the aging process. This intricate topic, with its distinctive characteristics and underlying mechanisms, has captured the attention of both clinical and fundamental research endeavors.

The immune system is a complex network composed of immune cells, immune microenvironment, and circulatory factors. A properly functioning immune system helps the body defend against external attacks and monitor internal damage and mutations^1^. As we traverse the journey of aging, profound transformations encompass every facet of our immune system. Recent groundbreaking studies reveal that the chronological aging of immune cells could, in fact, serve as a pivotal precursor to the overall aging process in the human body^2^. The immune system comprises two principal divisions: innate immunity and adaptive immunity. Of these, the latter has garnered significant attention in contemporary research, particularly focusing on the pivotal role of T cells, which are integral to the adaptive immune response, in the phenomenon of cellular senescence and its far-reaching consequences^1^, ^3^, ^4^. Currently, a comprehensive grasp of the aging dynamics within the innate immune system is lacking^5^, and there are few studies on the role of innate immune cell aging in promoting the aging of the whole immune system and even the whole body.

Neutrophils, constituting the most numerously prevalent immune effector cells within the circulatory system, play a pivotal role in innate immunity. These versatile cells boast an array of cytological capabilities, including chemotaxis, phagocytosis, degranulation, generation of reactive oxygen species (ROS), and the formation of neutrophil extracellular traps (NETs)^6^, ^7^. Neutrophils are indispensable players in the realms of host defense, immune modulation, tissue integrity, and even tumor progression^8^-^13^. Despite limited understanding of neutrophil senescence in both clinical and fundamental research contexts, our dedicated team has embarked on extensive investigations into the physiology and pathophysiology of neutrophils. Our focus lies in elucidating the mechanisms by which neutrophils impact the progression of sepsis, specifically uncovering the intricacies of their chemotactic role during severe infections and sepsis. Furthermore, we have illuminated the significance of neutrophil-induced immunosuppression in the development of organ dysfunction within the sepsis paradigm. We have also expanded our exploration to unravel the diversity and function of neutrophils within other disease models^14^-^17^. This current research endeavor delves into the intricate biological functioning and unique metabolic traits of aged neutrophils, and subsequently establishes a correlation between lipid metabolism and the age-linked cGAS-STING pathway. By doing so, it aims to pave the way for innovative interventions targeting aged neutrophils, thus opening up novel therapeutic perspectives.

## Materials and Methods

### Ethics and mouse model

This study was approved by the Medical Ethical Committee of Suzhou Municipal Hospital (animal ethical approval No. KL901390). All experiments followed approved guidelines. Male wildtype C57BL/6 mice aged 8 to 10 weeks and 22 to 24 months were utilized. They were housed in a pathogen-free environment and had free access to the same food and water.

### Mouse neutrophil isolation

The femurs and tibias of mice were isolated and rinsed with Hank's Balanced Salt Solution (HBSS) (calcium and magnesium-free, Gibco, USA) containing 2 mM Ethylenediaminetetraacetic acid (EDTA) (Thermo, USA) and 10% Fetal Bovine Serum (FBS) (Gibco, USA). A 70 μm cell strainer was used for filtration and debris removal. The collected bone marrow cells were pelleted at 4°C, 500 × g for 7 min. Red blood cells were lysed in distilled water for 15 s. Neutrophils were enriched using the EasySepTM Mouse Neutrophil Enrichment Kit (STEMCELL, USA).

### Luminex multiplex cytokine analysis

According to the Assay kit of mouse cytokine 23-plex (Bio-RAD, USA), 50 μL of beads, standard substance, control substance, and sample were added sequentially and incubated at 800 rpm for 1 h. After washing, 50 μL of detection antibodies were added and incubated at 800 rpm at room temperature for 1 h. After washing again, 50 μL of PE-coupled streptavidin was added and incubated for 30 min at 800 rpm at room temperature. Subsequently, 100-150 μL of sheath solution was added and incubated at 800 rpm at room temperature for 0.5-2 min. The samples were analyzed using the Luminex-200 system.

### Flow cytometry

To assess the post-activation surface molecule expression of neutrophils, cells were co-incubated on ice with 10 nM Phorbol 12-myristate 13-acetate (PMA) (Solarbio, China) and fluorescent antibodies for 30 min. For intracellular reactive oxygen species (ROS) detection in neutrophils, 10 μM 2',7'-Dichlorodihydrofluorescein diacetate (H2DCFDA) (Solarbio, China) was utilized. CD63 (BD, USA) was employed to assess neutrophil degranulation, while SYTOX Green Nucleic Acid Stain (Thermo, USA) was used to detect NETs and nuclear morphology. To assess the phagocytic capability of neutrophils, a Phagocytosis Assay (Red E. coli, Abcam, UK) was employed. Neutrophils at concentrations of 1 × 106/mL were co-cultured with red E. coli at 37°C/5% CO2 for 2 h. Samples were analyzed using a FACSC Canto II flow cytometer (BD Biosciences, USA) and data were processed with FlowJo V10.

### Laser scanning confocal microscopy

Mitochondria were stained with 50 mM MitoTracker Deep Red FM (AbMole BioScience, USA) for 2 hours at 37°C. Cell nuclei and neutrophil extracellular traps (NETs) were stained using 0.1 μg/mL 4',6-diamidino-2-phenylindole (DAPI) (Beyotime, China). Neutrophils were visualized using an LSM900 confocal laser scanning microscope (ZEISS, Germany).

### Transmission electron microscopy (TEM)

Neutrophils were sequentially fixed with 2.5% glutaraldehyde (Sigma-Aldrich) for 1 h and 2% osmium tetroxide for 2 h. After washing with double-distilled water, the cell samples were stained with 0.5% uranyl acetate for 12 h, dehydrated through a graded ethanol series, embedded in epoxy resin, and sectioned into 70-90 nm ultrathin sections. Images were acquired using a Tecnai G2 TWIN transmission electron microscope (FEI, USA).

### Under-agarose neutrophil chemotaxis model

The under-agarose neutrophil chemotaxis model was established as previously described^18^. Briefly, a 1.2% (w/v) agarose solution was prepared, boiled, and mixed in a 1:3 ratio with prewarmed medium containing 50% HBSS (with Ca2+ and Mg2+) and 50% RPMI 1640 supplemented with 20% heat-inactivated FBS. Approximately 2.7 mL of this mixture was poured into a 35-mm culture dish pre-chilled to 4°C. After 30 minutes of solidification, three wells (3 mm in diameter, 2.8 mm apart) were punched in a straight line in the gel. The central well was filled with 10 μL of chemoattractant WKYMVm (0.1 μmol/L, a potent neutrophil chemotactic peptide) (Abcam, USA), while the side wells were each filled with 10 μL of neutrophil suspension (1.0 × 10^7^ cells/mL, treated as indicated). Gels were incubated for 4 h in a 37°C/5% CO2 incubator. Neutrophil chemotaxis patterns were observed and documented using an Olympus IX71 inverted microscope under 4× magnification.

### Percoll isolation of neutrophils and the cell counter to infer percentage of absolute low density neutrophils (LDN) counts

Neutrophils were isolated from mouse femurs and tibias and enriched using the EasySep™ Mouse Neutrophil Enrichment Kit (STEMCELL Technologies, USA). The bone marrow polymorphonuclear (PMN) cell suspension was layered over a discontinuous Percoll (Sigma-Aldrich) gradient and centrifuged at 1000 × g for 35 min at 22°C, with no acceleration or brake. Cells at the 55-65% interface were collected as the low-density neutrophil (LDN) fraction, while cells at the 65-80% interface were collected as the high-density neutrophil (HDN) fraction. Cell pellets were resuspended in 1 mL of 1× PBS. A cell counter was used to determine the concentration of cells in each fraction, allowing calculation of the total number and percentage of LDNs and HDNs.

### Lipid extraction

For lipid extraction, 1 × 10^7^ neutrophils were homogenized in 200 μL water containing 20 μL of internal lipid standard mixture. The homogenate was mixed with 800 μL of methyl tert-butyl ether (MTBE) and 240 μL of pre-cooled methanol by vortexing. The mixture was sonicated in a low-temperature water bath for 20 minutes, then incubated at room temperature for 30 min. After centrifugation at 14,000 × g for 15 min at 10°C, the upper organic phase was collected and dried under a stream of nitrogen gas. For mass spectrometry analysis, the dried lipid extract was reconstituted in 200 μL of 90% isopropanol/acetonitrile solution. After thorough vortexing, 90 μL of the reconstituted sample was centrifuged at 14,000 × g for 15 min at 10°C, and the supernatant was used for injection.

### Untargeted lipidomics

Samples were separated using an UHPLC Nexera LC-30A ultra-high performance liquid chromatography system equipped with a C18 column. The column temperature was maintained at 45°C, and the flow rate was set at 300 μL/min. The mobile phase consisted of solvent A (acetonitrile:water, 6:4, v/v) and solvent B (acetonitrile:isopropanol, 1:9, v/v). The gradient elution program was as follows: 0-3.5 min, 40% B; 3.5-13 min, 40-75% B; 13-19 min, 75-99% B; 19-24 min, 40% B. Samples were kept at 10°C in an autosampler throughout the analysis. To minimize the impact of instrument signal fluctuations, samples were analyzed continuously in random order.

Both positive and negative ion modes of electrospray ionization (ESI) were employed for detection. The UHPLC-separated samples were analyzed using a Q Exactive series mass spectrometer (Thermo Scientific™). ESI source parameters were set as follows: heater temperature, 300°C; sheath gas flow rate, 45 arbitrary units; auxiliary gas flow rate, 15 arbitrary units; sweep gas flow rate, 1 arbitrary unit; spray voltage, 3.0 kV; capillary temperature, 350°C; S-Lens RF level, 50%. The MS1 scan range was set to 200-1800 m/z for lipid molecules and fragments. For each full scan, ten data-dependent MS2 scans were acquired using higher-energy collisional dissociation (HCD). The resolution was set to 70,000 for MS1 and 17,500 for MS2 at m/z 200.

### Lipidomic data analyses

Lipidomic data were analyzed using MetaboAnalyst 5.0 and the LINT-web platform. Briefly, the data were normalized to the median value, log-transformed, and auto-scaled. The relative abundance of individual metabolites/lipids and the mean abundance of ceramides were then calculated based on the processed data.

### Assessment of Mitochondrial Permeability Transition Pore (MPTP) opening

Mitochondrial Permeability Transition Pore (MPTP) opening was assessed using an MPTP Assay Kit (Beyotime, China). Briefly, treated cells were washed with phosphate-buffered saline (PBS) and incubated with calcein AM plus Co2+ quencher at 37°C for 30 min. After two additional PBS washes, the residual fluorescence intensity was analyzed by flow cytometry.

### Quantitative Reverse Transcription-Polymerase Chain Reaction (qRT-PCR)

Total RNA was isolated using TRIzol reagent (Thermo Fisher Scientific). Extracted RNA (500 ng) was reverse-transcribed into cDNA using the PrimeScript™ RT reagent Kit (Takara, Japan). Quantitative real-time PCR was performed using an Applied Biosystems QuantStudio 5 system and SYBR Green PCR Master Mix (Takara, Japan). Relative gene expression was determined by comparing target gene expression to that of the reference gene GAPDH. The following primer sequences were used: GAPDH forward: 5'-AAGGGCTCATGACCACAGTC-3', reverse: 5'-CAGGATGATGTTCTGGGCA-3'; STING forward: 5'-GGTCACCGCTCCAAATAGTAG-3', reverse: 5'-CAGTAGTCCAAGTTCGTGCG-3'; IFN-β forward: 5'-CGTGGGAGATGTCCTCAACT-3', reverse: 5'-CCTGAAGATCTCTGCTCGGAC-3'.

### Western blot (WB)

Cellular samples were lysed in ice-cold RIPA buffer supplemented with phosphatase and protease inhibitors (Sigma, USA) for 30 min. Lysates were mixed with 5× loading buffer (Beyotime, China) at a 4:1 ratio and boiled for 6 min. Proteins were separated by SDS-PAGE using 10-12.5% gradient gels and transferred onto PVDF membranes using a GE Healthcare transfer system. Membranes were blocked with 5% skim milk for 1 h, then incubated overnight with primary antibodies at 4°C, followed by a 2h incubation with appropriate HRP-conjugated secondary antibodies at room temperature. The following primary antibodies were used (all at 1:1000 dilution unless otherwise stated): anti-cleaved-caspase3 (Affinity Bioscience, AF6311); anti-caspase3 (Affinity Bioscience, AF7022); anti-Bcl-2 (Affinity Bioscience, AF6139); anti-Bax (Affinity Bioscience, AF0120); anti-GAPDH (Abways, AB0037, 1:5000); anti-p-TBK1(Ser172) (Affinity Bioscience, AF8190); anti-TBK1 (Affinity Bioscience, DF7026); anti-p-STING(Ser366) (Affinity Bioscience, AF7416); anti-STING (Affinity Bioscience, DF12090). Secondary antibodies were HRP-conjugated goat anti-rabbit IgG (H+L) (Proteintech, SA00001-2) and HRP-conjugated goat anti-mouse IgG (H+L) (Proteintech, SA00001-1). Protein bands were visualized using an enhanced chemiluminescence reagent (Thermo Fisher Scientific, USA).

### Statistical analysis

All statistical analyses and graphical representations were performed using GraphPad Prism 8.0 software. The Shapiro-Wilk test was used to assess the normality of continuous variables. Results are expressed as mean ± standard deviation (SD). For comparisons between multiple groups, one-way analysis of variance (ANOVA) was used for normally distributed variables, followed by Tukey's post-hoc test or Dunn's post-hoc test for multiple comparisons. Student's t-test or Wilcoxon matched-pairs signed rank test was applied to compare differences between two groups. Statistical significance was set at p < 0.05. P values are represented as follows: NS (not significant), *p < 0.05, p < 0.01, and* p < 0.001.

## Results

### Systemic oxidative stress levels were elevated in aged mice compared to young mice

**Figure 1:**
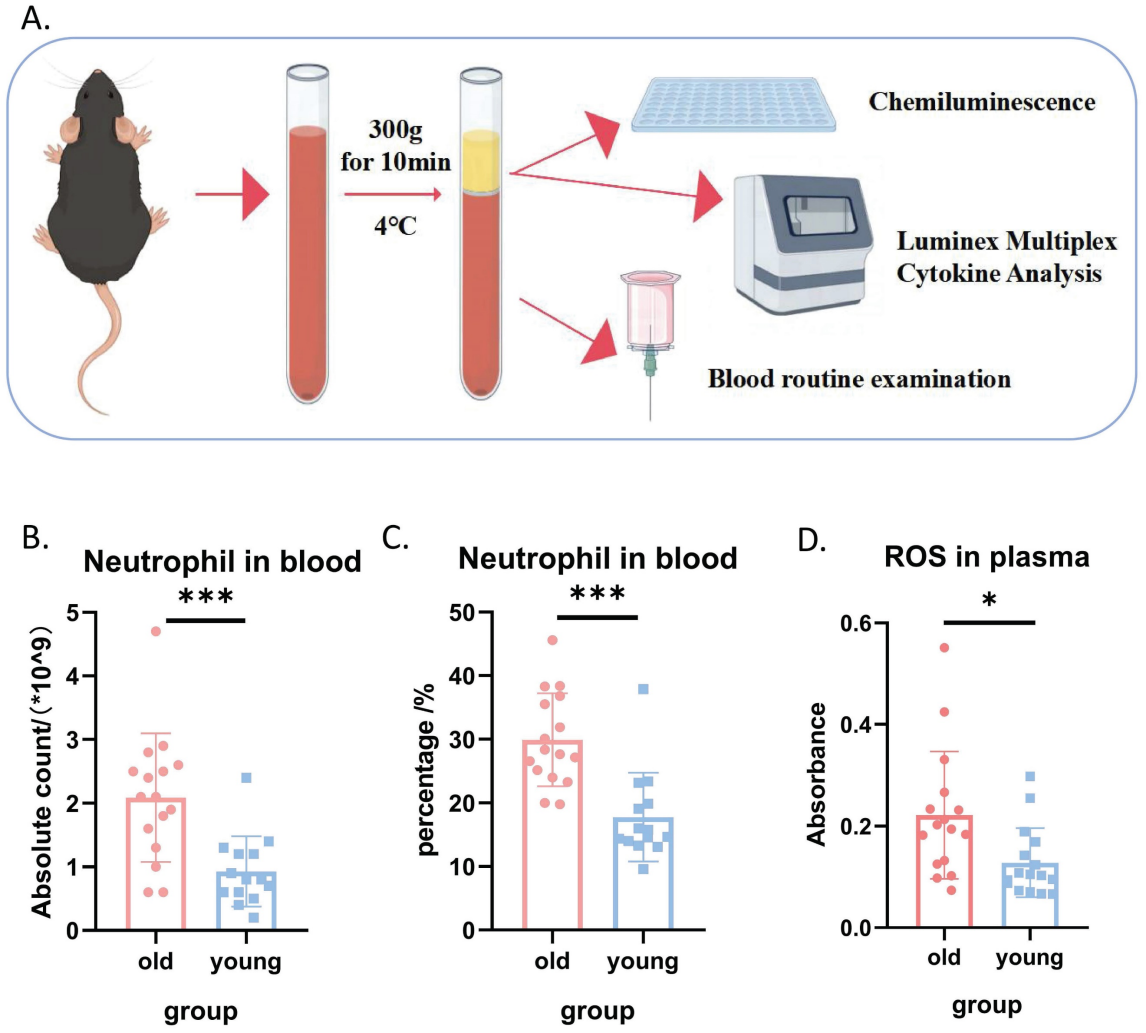
** Systemic oxidative stress levels were elevated in aged mice compared to young mice.** (A) Schematic diagram illustrating the experimental design for comparing systemic inflammatory markers in young and aged mice. (B) Absolute neutrophil count in peripheral blood (n=14-16 per group). (C) Percentage of neutrophils in peripheral blood (n=14-16 per group). (D) ROS levels in peripheral blood plasma (n=14-16 per group).

Given the well-established link between oxidative stress and the aging process, we first compared systemic oxidative stress levels in young and aged mice, as illustrated in **Figure 1A**. Complete blood count analysis revealed that both the absolute count and percentage of neutrophils in the peripheral blood of aged mice were significantly higher than those in young mice (**Figures 1B and 1C**). Furthermore, plasma reactive oxygen species (ROS) levels were markedly elevated in aged mice compared to young mice (**Figure 1D**), collectively indicating a higher systemic oxidative stress burden in aged mice. Interestingly, we observed no significant differences in plasma levels of several common inflammatory factors between the two groups (**Supplemental Figures S1-S2**). This lack of difference in inflammatory markers is consistent with the expectation that immune cells primarily secrete cytokines in response to stimuli, rather than constitutively.

### Aged neutrophils exhibited significantly impaired biological functions

**Figure 2:**
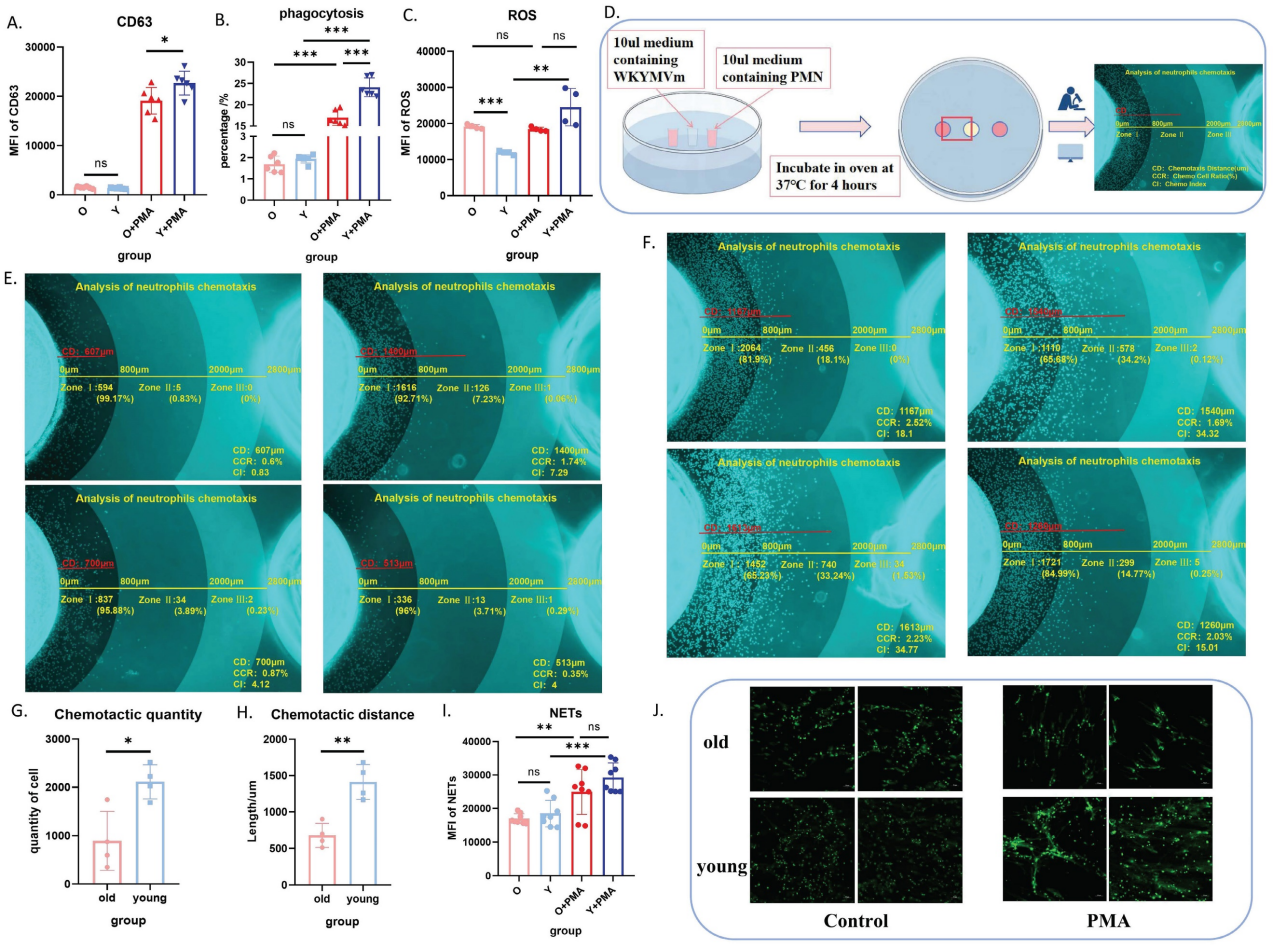
** Aged neutrophils exhibit significantly impaired biological functions.** (A) Flow cytometric analysis of CD63 expression in neutrophils following PMA stimulation or no stimulation (n=6). (B) Flow cytometric quantification of phagocytosis after co-incubation of neutrophils with fluorescent E. coli analogues at 37°C for 2 h (n=6). (C) Flow cytometric measurement of ROS levels using DCFH-DA staining after neutrophil stimulation with PMA at 37°C for 30 min (n=4). (D) Schematic representation of the murine neutrophil agarose chemotaxis model. (E) Representative images of aged neutrophil chemotaxis after 4 h incubation at 37°C in an agarose chemotaxis model (n=4). (F) Representative images of young neutrophil chemotaxis after 4 h incubation at 37°C in an agarose chemotaxis model (n=4). (G) Quantification of chemotactic cell numbers in the mouse neutrophil chemotaxis model (n=3). (H) Measurement of chemotactic distance in the mouse neutrophil chemotaxis model (n=3). (I-J) Analysis of NET formation: neutrophils were stimulated with PMA at 37°C for 4 h, followed by flow cytometric detection of fluorescent expression after SYTOX staining, and visualization of NET morphology using confocal microscopy (n=8). PMN: polymorphonuclear neutrophil; PMA: phorbol 12-myristate 13-acetate; NETs: neutrophil extracellular traps.

Our previous study demonstrated no significant functional differences between bone marrow-derived and peripheral blood-derived neutrophils^19^. Therefore, we isolated bone marrow neutrophils using magnetic beads for subsequent functional analyses. We concurrently evaluated the biological functions of purified neutrophils with and without *in vitro* PMA stimulation, including degranulation (**Figure 2A**), phagocytosis (**Figure 2B**), ROS production (**Figure 2C**), chemotaxis (**Figure 2D-H**), and NET formation (**Figure 2I-J**). These comprehensive assessments revealed impaired baseline functions and PMA responsiveness in bone marrow-derived neutrophils from aged mice, with a propensity for elevated oxidative stress responses.

### Significant heterogeneity in neutrophil populations between aged and young mice

To identify potential targets for interfering with neutrophil senescence, we investigated the heterogeneity of neutrophils in the senescent state. For experimental consistency and comparability, neutrophils were purified using magnetic beads. We observed a significantly higher proportion of low-density neutrophils (LDN) in aged mice compared to young mice (**Supplemental Figure S3A, B**)**.** Given the known immunosuppressive properties of LDN, we further examined the expression of immunosuppressive markers ARG-1 and PD-L1, finding increased expression on neutrophils from aged mice (**Supplemental Figure S3C, D**).

**Figure 3:**
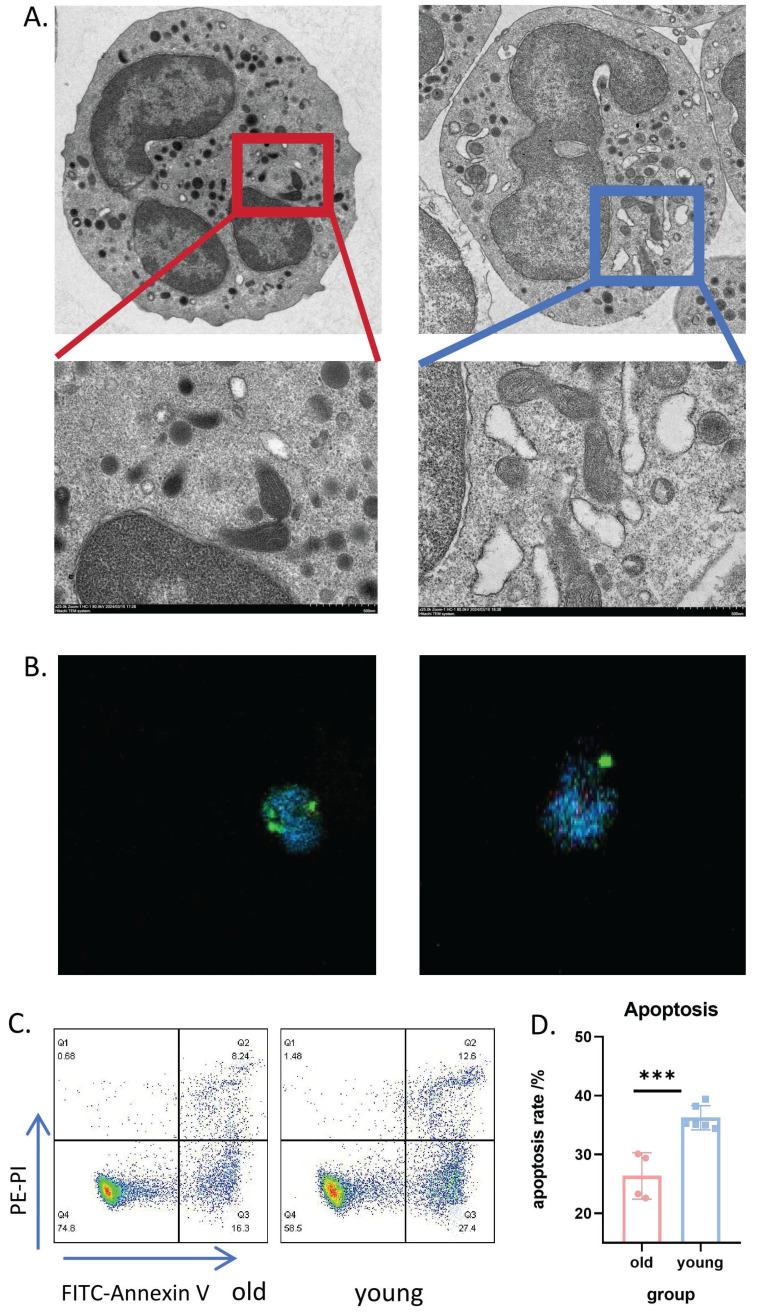
** Significant heterogeneity in neutrophil populations between aged and young mice.** (A) Transmission electron microscopy images showing representative microstructure of bone marrow-derived neutrophils from aged (left) and young (right) mice (n=3). (B) Immunofluorescence confocal imaging showing the intracellular structure of neutrophils in aged (left) and young (right) mice: green (cell membrane), blue (nucleus), red (mitochondria) (n=3). (C) Representative flow cytometry scatter plots showing expression of apoptosis-related Annexin V-PI in bone marrow neutrophils from aged and young mice after 4 hours of *in vitro* culture. (D) Flow cytometric quantification of apoptosis levels in neutrophils from aged and young mice cultured *in vitro* for 4h (n=4-6). LDN: Low Density Neutrophils; HDN: High Density Neutrophils.

Transmission electron microscopy of neutrophils from aged mice in the basal state revealed dense cytoplasm and clumped or concentrated nuclear chromatin, with chromatin edges concentrated on the inner side of the nuclear envelope. The number of mitochondria and endoplasmic reticulum was significantly reduced, with many showing obvious pyknosis, swelling, and hydrops (**Figure 3A, left**). In contrast, neutrophils from young mice exhibited a significantly higher number of mitochondria with normal ultrastructural morphology and clearly visible mitochondrial microstructure (**Figure 3A, right**). Immunofluorescence confocal microscopy further confirmed the reduced number of mitochondria in PMNs from aged mice (**Figure 3B and Supplemental videos S1-6**).

We also examined the membrane expression levels of chemotaxis-related receptors CXCR2, CXCR4, and P2RX1 (**Supplemental Figure S3E-G**). Notably, CXCR4 and P2RX1 levels were significantly upregulated in neutrophils from aged mice. After 4 hours of *in vitro* culture, neutrophils from aged mice demonstrated a significant delay in apoptosis (**Figure 3C-D**). Collectively, these experiments indicate remarkable heterogeneity in senescent neutrophils.

### Distinct metabolic patterns in neutrophils from aged and young mice

Since senescent neutrophils are obviously heterogeneous and cannot effectively play biological functions under stress, and may even cause tissue damage and systemic inflammation through high levels of oxidative stress, we believe that the core of intervention on neutrophil senescence is to inhibit the delay of apoptosis of senescent neutrophils and eliminate senescent neutrophils.

In our previous study, we found that delayed apoptosis of neutrophils was associated with altered metabolic patterns^20^. Therefore, we hypothesized that the shift in PMN metabolic pattern may be an important factor in the induction of senescence.

**Figure 4:**
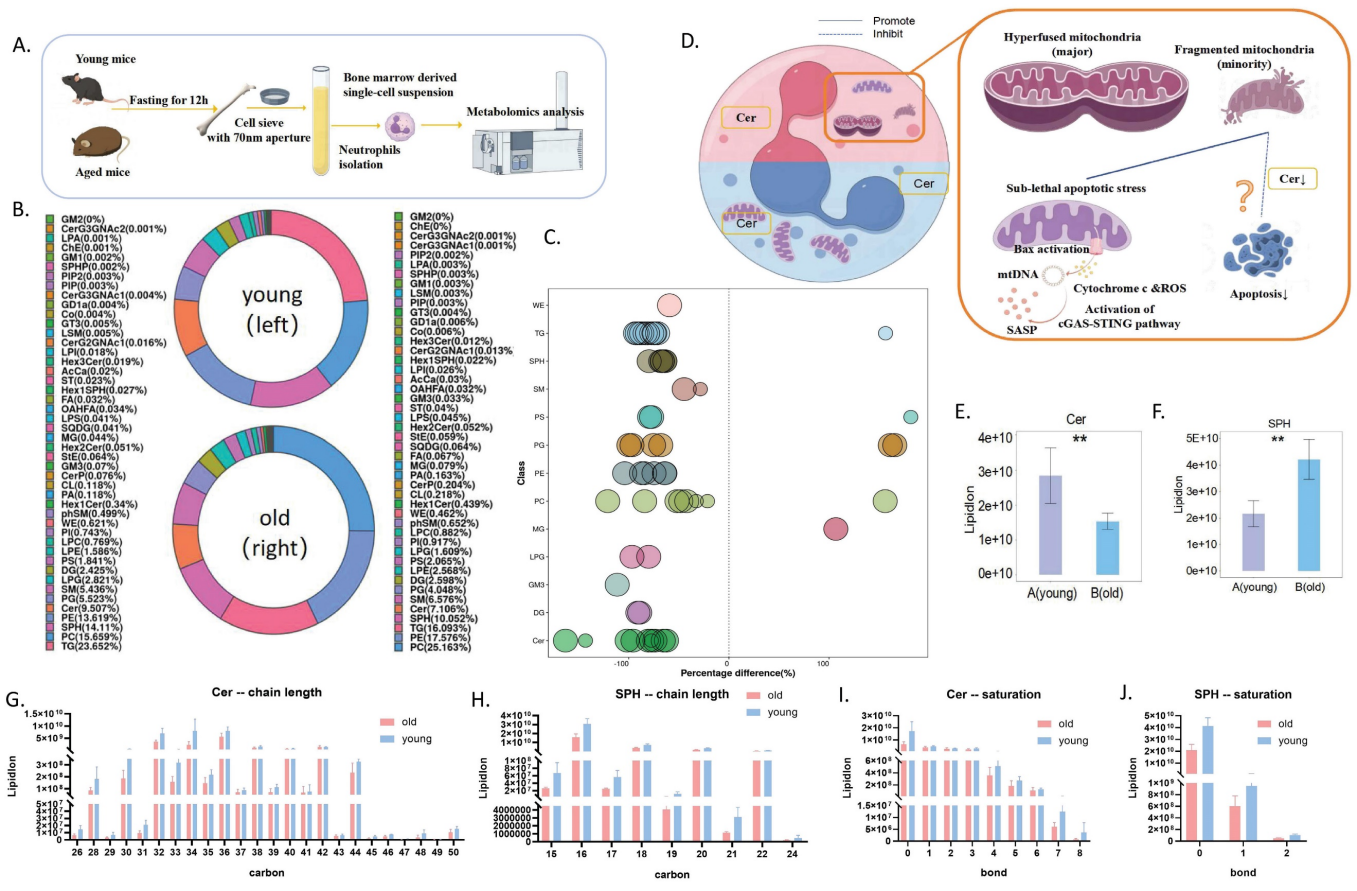
** Distinct metabolites in neutrophils from aged and young mice.** (A) Schematic diagram of neutrophil metabolomics workflow: young and old mice were sacrificed after respiratory anesthesia under uniform feeding conditions, and neutrophils were purified by magnetic beads for metabolomics detection and analysis. (B) Lipid composition in bone marrow-derived neutrophils from aged and young mice (A=young mice; B=aged mice) (n=7). (C) Metabolomics bubble plot: Bubbles represent significantly different lipid molecules, with bubble size indicating the significance of the difference. Smaller bubbles represent significant differences (0.01 < p-value < 0.05), while larger bubbles indicate highly significant differences (p-value < 0.01). (D) Model diagram hypothesizing the involvement of Cer in neutrophil senescence and delayed apoptosis. (E) Ceramide (Cer) levels in bone marrow-derived neutrophils from aged and young mice (A=young mice; B=aged mice) (n=7). (F) Sphingosine (SPH) levels in neutrophils from aged and young mice (A=young mice; B=aged mice) (n=7). (G) Ceramide chain length analysis. (H) Sphingosine chain length analysis. (I) Ceramide unsaturation analysis. (J) Sphingosine unsaturation analysis.

**Figure 5:**
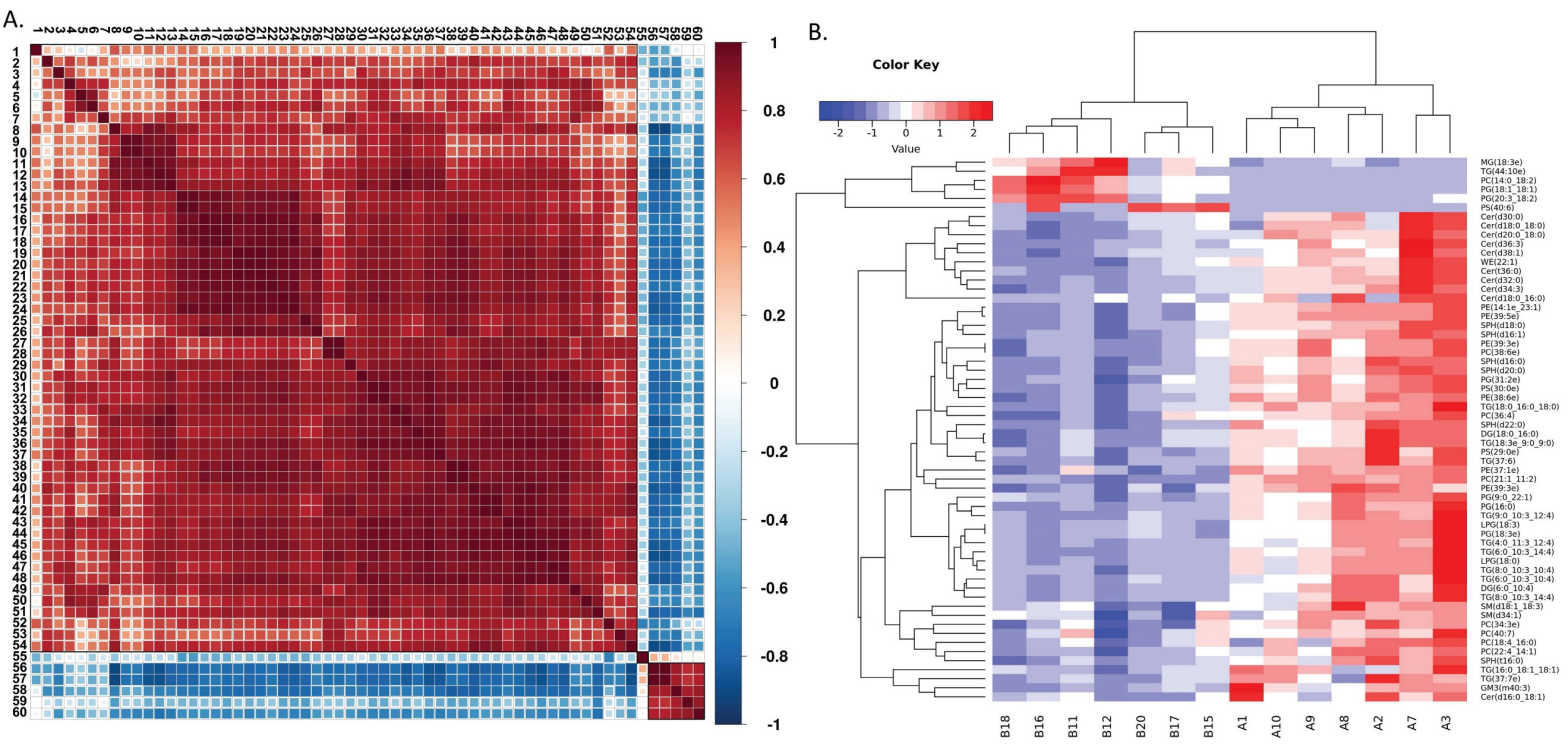
** Distinct metabolic patterns in neutrophils from aged and young mice.** (A) Correlation clustering heat map analysis of significantly different lipids (A=young mice; B=aged mice) (n=7). (B) Hierarchical clustering analysis of lipids with significant differences between groups (A=young mice; B=aged mice) (n=7). The metabolites in Figure 5(A) are represented by numbers: 1:Cer(18:0/16:0);2:Cer(16:0/18:1);3:TG(16:0/18:1/18:1);4:PC(40:7);5:SM(34:1);6:SM(18:1/18:3);7:PC(18:4/16:0);8:Cer(36:3);9:Cer(30:0);10:Cer(18:0/18:0);11:Cer(38:1);12:Cer(20:0/18:0);13:Cer(36:0);14:PG(18:3);15:LPG(18:3);16:TG(8:0/10:3/14:4);17:TG(6:0/10:3/10:4);18:DG(6:0/10:4);19:TG(8:0/10:3/10:4);20:TG(6:0/10:3/14:4);21:LPG(18:0);22:TG(4:0/11:3/12:4);23:PG(16:0);24:TG(9:0/10:3/12:4);25:PC(21:1/11:2);26:PG(9:0/22:1);27:PC(38:6);28:PE(39:3);29:SPH(16:0);30:WE(22:1);31:PE(14:1/23:1);32:PE(39:5);33:Cer(32:0);34:Cer(34:3);35:PC(36:4);36:SPH(18:0);37:TG(18:0/16:0/18:0);38:PS(29:0);39:TG(37:6);40:SPH(22:0);41:TG(18:3/9:0/9:0);42:DG(18:0/16:0);43:SPH(16:1);44:SPH(16:0);45:SPH(20:0);46:PE(38:6);47:PG(31:2);48:PS(30:0);49:PC(22:4/14:1);50:PC(34:3);51:PE(39:3);52:PE(37:1);53:TG(37:7);54:GM3(40:3);55:PS(40:6);56:PG(18:1/18:1);57:PC(14:0/18:2);58:MG(18:3e);59:PG(20:3/18:2);60:TG(44:10).

Building on this, we compared the metabolomic profiles of neutrophils from aged and young mice (**Figure 4A**). Although the overall lipid composition was generally similar between the two groups (**Figure 4B**), neutrophils from aged mice exhibited distinct lipid metabolism patterns, as revealed by correlation clustering heat map analysis of significantly different lipids (**Figure 5A**), hierarchical clustering analysis (**Figure 5B**), and comparison of statistically significant lipid molecules (**Figure 4C**).

By analyzing the differential metabolites of neutrophil metabolomics in aging mice, we found that ceramide may be of significant significance. Notably, we observed significantly decreased levels of both ceramide (Cer) and its metabolite sphingosine (SPH) in aged mice (**Figure 4E-F**), independent of chain length and saturation (**Figure 4G-J**). Moreover, ceramide has been shown to regulate apoptosis^21^. Therefore, we targeted ceramide as a candidate target for further exploration of aging mechanisms (**Figure 4D**).

### Ceramide mixture promotes apoptosis and improves biological functions of neutrophils in aged mice

Given that ceramide levels of varying chain lengths and saturation were significantly lower in neutrophils from aged mice compared to young mice (**Figure 4G-J**), we selected an exogenously added ceramide mixture for intervention. We first sought to determine the optimal concentration of this ceramide mixture that could eliminate aged neutrophils while minimizing effects on young neutrophils. Our preliminary experiments revealed that, in contrast to the conventional 1 μg/mL ceramide concentration used to promote neutrophil apoptosis, a 2 mg/mL ceramide concentration significantly inhibited apoptosis in neutrophils from young mice (**Supplemental Figure S4**). This higher concentration was thus selected as the optimal intervention dose.

**Figure 6:**
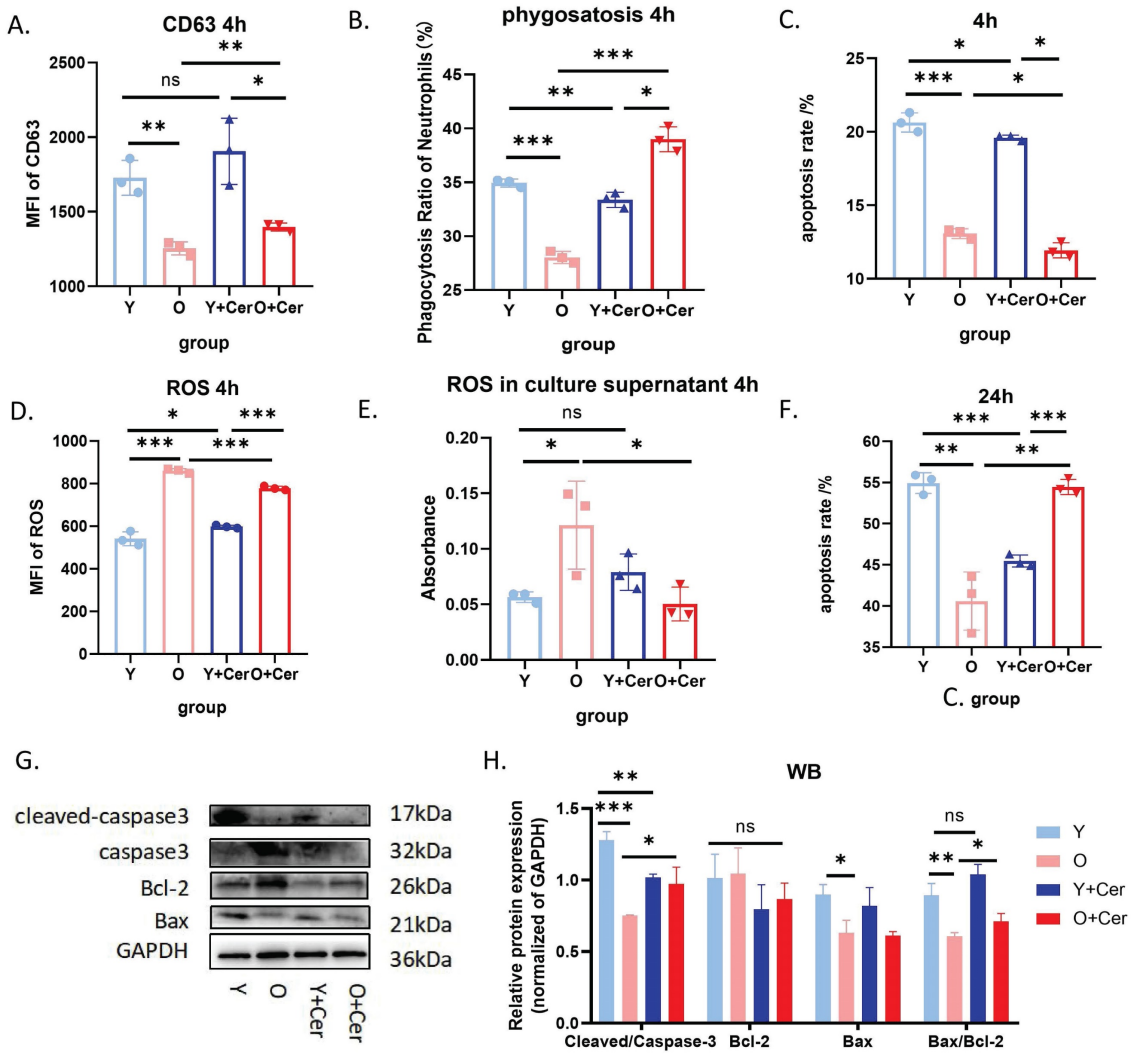
***In vitro* intervention with ceramide mixture promotes apoptosis and improves biological functions of neutrophils in aged mice.** (A) Flow cytometry analysis of CD63 expression on neutrophils treated with ceramide for 4h (and without intervention) (n=3). (B) Flow cytometry analysis of phagocytosis ratio after co-incubation of ceramide-treated (4h) or untreated neutrophils with fluorescent E. coli analogues at 37°C for 2 h (n=3). (C) Flow cytometry analysis of apoptosis levels in neutrophils from aged and young mice cultured with ceramide *in vitro* for 4h (n=3). (D) Flow cytometry analysis with DCFH-DA staining showing ROS levels after neutrophil culture with ceramide *in vitro* for 4h (n=3). (E) ROS probe detection of ROS levels in culture supernatants of neutrophils treated with ceramide for 4h (and without treatment) (n=3). (F) Flow cytometry analysis of apoptosis levels in neutrophils from aged and young mice cultured with ceramide *in vitro* for 24h (n=3). (G-H) Protein abundance of apoptosis-related proteins in neutrophils with representative virtual blot bands (n=3).

Building on these findings, flow cytometry results demonstrated that after 24 hours of *in vitro* culture, the apoptosis level of neutrophils in aged mice was significantly lower than in young mice. Intervention with 2 mg/mL ceramide for 24 hours significantly increased apoptosis in neutrophils from aged mice while delaying apoptosis in neutrophils from young mice (**Figure 6F**). Western blot analysis of apoptosis-related proteins corroborated these flow cytometry results (**Figure 6G-H**). Based on these data, we selected 2 mg/mL as the optimal intervention concentration for the ceramide mixture.

We further evaluated the effects of *in vitro* intervention with 2 mg/mL ceramide mixture on neutrophil biological functions. To avoid the confounding effects of high apoptosis rates at 24 hours, we opted for a 4-hour intervention period for these functional assessments. Our study revealed that after 4 hours of *in vitro* culture under basal conditions, neutrophil degranulation levels in aged mice were significantly lower than in young mice. Ceramide intervention for 4 hours significantly increased degranulation levels in aged mice, with no significant change observed in young mice (**Figure 6A**).

Similarly, the phagocytic capacity of neutrophils from aged mice was significantly lower than that of young mice after 4 hours of *in vitro* culture under basal conditions. The 4-hour ceramide intervention significantly increased the phagocytic capacity of neutrophils from aged mice (**Figure 6B**). Regarding reactive oxygen species (ROS) production, baseline intracellular ROS levels in neutrophils from aged mice were significantly higher than in young mice. However, ceramide intervention for 4 hours significantly decreased ROS levels in aged mice (**Figure 6D**).

ROS levels in neutrophil culture supernatants from aged mice were higher than those from young mice after 4 hours of *in vitro* culture under basal conditions. Ceramide intervention for 4 hours decreased ROS levels in supernatants from aged mice, while changes in supernatants from young mice were not statistically significant (**Figure 6E**). Collectively, these results demonstrate that ceramide intervention *in vitro* can significantly improve the biological functions of neutrophils from aging mice and reduce oxidative stress levels.

Interestingly, we also observed that after 4 hours of ceramide intervention, apoptosis was delayed in neutrophils from both young and old mice (**Figure 6C**). Given that neutrophil activation is associated with delayed apoptosis, we hypothesize that high ceramide concentrations may activate neutrophils in aged mice, thereby improving their functionality. Moreover, we demonstrated that 24 hours after intervention, neutrophil apoptosis was significantly increased in aged mice without causing neutrophil accumulation and subsequent damage.

In conclusion, *in vitro* treatment with 2 mg/mL ceramide mixture can inhibit delayed apoptosis in senescent neutrophils, improve their biological functions, and reduce oxidative stress levels. These findings suggest that ceramide could serve as an important target for interventions aimed at mitigating neutrophil senescence.

### Ceramide interferes with neutrophil senescence through the cGAS-STING pathway

**Figure 7:**
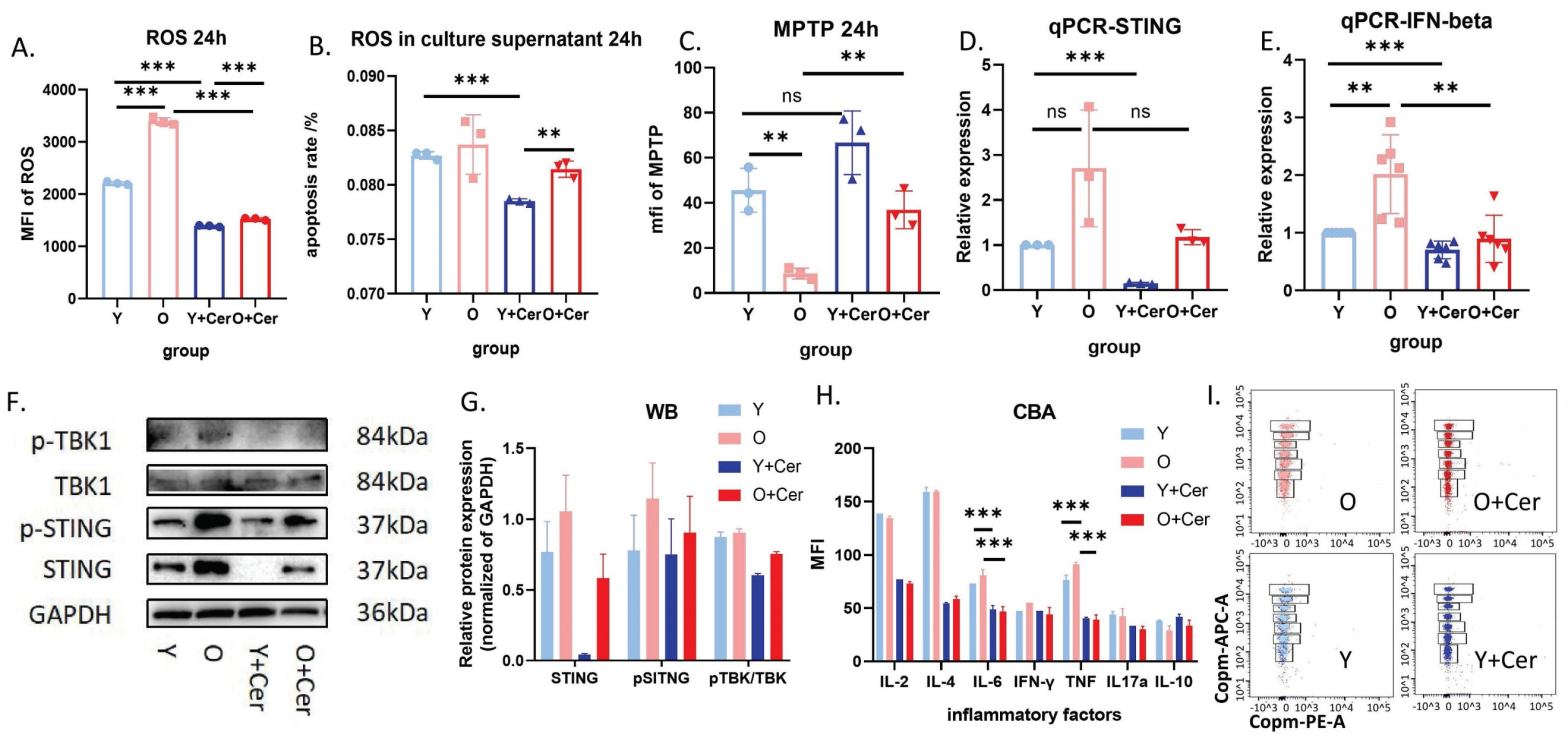
** Ceramide interferes with neutrophil senescence through the cGAS-STING pathway.** (A) Flow cytometry analysis with DCFH-DA staining showing ROS levels after neutrophil culture with ceramide *in vitro* for 24h (n=3). (B) ROS probe detection of ROS levels in culture supernatants of neutrophils treated with ceramide for 24h (and without treatment) (n=3). (C) Flow cytometry analysis of mitochondrial permeability transition pore opening in neutrophils (n=3). (D) qPCR analysis of STING gene expression in neutrophils treated with ceramide for 24h (and without intervention) (n=3). (E) qPCR analysis of IFN-beta gene expression in neutrophils treated with ceramide for 24h (and without intervention) (n=3). (F) Western blot analysis of protein expression levels in the cGAS-STING-TBK pathway in neutrophils treated with ceramide for 24h (and without intervention) (n=3). (G) Statistical analysis of Western blot protein expression levels (n=3). (H) Flow cytometry analysis of inflammatory factor levels in culture supernatants of neutrophils treated with ceramide for 24h (and without intervention) (n=2). (I) Representative FACS plots from CBA showing levels of inflammatory factors in culture supernatants of neutrophils treated with ceramide for 24h (and without intervention).

After 24 hours of *in vitro* culture under basal conditions, intracellular ROS levels in neutrophils from aged mice were significantly higher than in young mice. Ceramide intervention for 24 hours significantly decreased ROS levels in neutrophils from both groups (**Figure 7A**). Interestingly, there was no significant difference in ROS levels in neutrophil culture supernatants from aged and young mice after 24 hours of *in vitro* culture under basal conditions. However, ceramide intervention for 24 hours significantly decreased ROS levels in supernatants from both groups (**Figure 7B**).

The mitochondrial permeability transition pore opening in neutrophils from aged mice cultured *in vitro* for 24 hours was significantly higher than in young mice. After 24 hours of ceramide intervention, mitochondrial permeability in neutrophils from aged mice decreased significantly, while no significant difference was observed in young mice (**Figure 7C**). These results suggest that ceramide improved mitochondrial permeability in neutrophils from aged mice, leading to reduced intracellular ROS levels and decreased ROS release into the culture supernatant.

Previous studies have reported that increased mitochondrial permeability can lead to mtDNA release and activation of the cGAS-STING pathway, which is closely associated with aging and inflammation. Therefore, we investigated this mechanism at both the RNA and protein levels. qPCR results showed that STING and IFN-beta expression levels in neutrophils from aged mice were higher than in young mice after 24 hours of *in vitro* culture, suggesting activation of the cGAS-STING pathway in aged mice. Notably, ceramide intervention downregulated this pathway (**Figure 7D-E**).

Western blot results were consistent with qPCR findings, indicating that STING and pSTING levels were higher in aged mice compared to young mice. Furthermore, phosphorylation levels of STING and TBK were significantly decreased in both groups after ceramide treatment (**Figure 7F-G**). Analysis of inflammatory factors in neutrophil culture supernatants revealed no significant differences between aged and young mice after 24 hours of *in vitro* culture under basal conditions. However, 24 hours of ceramide intervention significantly decreased levels of inflammatory factors IL-6 and TNF in both groups (**Figure 7H**). Representative images of FACS plots from CBA are shown in** Figure 7I**.

## Discussion

A striking observation in our study is the significantly augmented absolute count and percentage of circulating neutrophils in aged mice compared to their younger counterparts. Chemotaxis models and recognized related surface markers have demonstrated that neutrophils from aged mice have reduced chemotactic capacity and are more likely to be retained in the circulation. At the same time, delayed apoptosis and reduced clearance of neutrophils in aged mice also explain the phenomenon of increased circulating neutrophils. We further evaluated the biological functions of aged neutrophils and found that although there was no significant change in phagocytosis, degranulation and NETs formation ability under basal condition, the phagocytosis, degranulation and NETs formation and mobilization ability of PMN from aged mice were significantly reduced under stress condition. The level of cytoplasmic ROS in neutrophils of aged mice was significantly increased in the basal state, and neutrophils were an important source of circulating ROS, which was consistent with the high level of circulating ROS in aged mice. The increase of ROS will trigger oxidative stress, which in turn will cause DNA damage, cell dysfunction, and finally lead to aging^22^. Previous studies have shown that ROS directly contribute to paracrine induced senescence through intercellular communication via gap junctions^23^. However, PMN of aging mice cannot rapidly synthesize a large amount of ROS under stress. These results suggest that the anti-infective ability of PMNS accumulated in the circulation of aging mice is decreased, and the oxidative stress is increased in the resting state. In order to alleviate the negative effects of aging PMN, we tried to interfere with PMN senescence by promoting its apoptosis. Our previous study found that metabolic kinetics may be a key determinant of neutrophil apoptosis^20^. At the same time, studies have shown that changes in metabolic pathways significantly affect the function of neutrophils^24^. Based on the above conclusions, we propose the hypothesis that by artificially intervening PMN metabolism, the delay of apoptosis can be inhibited and neutrophil senescence may be improved.

We found a significant decrease in ceramide (Cer) and sphingosine (Sph) levels of PMNS from aging mice by metabolomics analysis. These molecules are thought to be negative regulators of cell proliferation and promote apoptosis^21^, ^24^. Interestingly, we found that specific concentrations of ceramide could improve the biological function of senescent PMN while promoting apoptosis. In contrast, higher doses of ceramide inhibited PMN apoptosis in young mice compared with conventional doses. Based on these results, we delved into the mechanism by which ceramide regulates neutrophil apoptosis. We found that *in vitro* intervention with ceramide improved mitochondrial membrane stability and inhibited cGAS-STING signaling. This in turn inhibited apoptosis and reduced the release of inflammatory cytokines. Recent studies have highlighted the critical role of small amounts of mitochondrial outer membrane permeability as a marker of cellular senescence, with the underlying mechanism associated with activation of the cGAS-STING pathway. Inhibition of mitochondrial outer membrane permeability can prolong the healthy life span of mice^24^. Our study showed that aged neutrophils exhibited a significant decrease in mitochondrial count after 24 h *in vitro*, accompanied by a slight increase in mitochondrial membrane permeability, which triggered a delay in apoptosis and exhibited typical aging features. Ceramide intervention *in vitro* can repair the mitochondrial membrane permeability of these senescent neutrophils, effectively inhibit the activation of cGAS-STING pathway, reduce the synthesis of pro-inflammatory factors, and thus alleviate the sening-related secretory phenotype. However, the inhibitory effect of ceramide on apoptosis was not evident in aged neutrophils and may be related to the significantly reduced number of mitochondria in aged neutrophils. Notably, the expression of Bcl-2, an apoptotic regulatory protein, was significantly upregulated, ultimately contributing to the apoptotic process in senescent neutrophils. Previous studies have shown that ceramide regulates apoptosis by regulating the expression and activity of apoptosis-related proteins and mitochondrial dynamics^21^, which is consistent with our experimental conclusion.

As an important mode of death of neutrophils under physiological conditions^25^, ^26^, apoptosis can not only prevent inflammation, but also prevent tissue damage through effective uptake by macrophages. In this study, we found that the process of PMN apoptosis was significantly prolonged in aged mice, leading to the accumulation of senescent cells. In addition to the normal physiological state, the phenomenon of delayed apoptosis is also prevalent in many pathological states, and these persistent neutrophils play an important role in the onset and progression of age-related diseases^27^-^29^. Thus, delayed apoptosis is a key feature that distinguishes PMN senescence and may provide insights into the treatment of aging-related diseases and identify potential intervention targets for the aging process. Recent studies have found that increasing apoptosis by regulating the neutrophil death pathway can reduce lung inflammation and injury, thus having great potential in the treatment of inflammatory diseases^30^. There is a correlation between the process of neutrophil senescence and systemic inflammatory state, immune function, and even aging phenotypes in aged mice. Therefore, the key challenge for future research is to design strategies to slow down neutrophil senescence and effectively eliminate elderly neutrophils, ultimately improving systemic inflammation, immune dysregulation, and aging-related diseases in the elderly population.

In this investigation, we revealed nuanced aspects of aged PMNs through examination of cellular functionality and inherent diversity. Moreover, we probed the underlying mechanism by which ceramides influence PMN senescence, primarily through modulation of apoptosis. The crux of this mechanism revolves around harmonizing the expression equilibrium of the apoptotic protein and the activation of the cGAS-STING signaling cascade.

This study introduces a novel perspective into the exploration of innate immunity aging and illuminates the intricate interplay between the immune system and organismal senescence, thereby establishing a solid foundation for future research delving into the systemic orchestration of senescent immune cells. However, our study also acknowledges certain limitations: (1) While the overarching role of ceramides in governing aged neutrophil apoptosis is established, the finer details of this regulatory process remain elusive. In subsequent phases, we plan to design experiments to further elucidate potential targets for intervention in neutrophil senescence. (2) Senescence is a systemic transformation, and while targeted manipulation of neutrophils can enhance innate immunity function and confer protective benefits, it might be insufficient to counteract the comprehensive effects of bodily aging. Consequently, further assessment is imperative, particularly to evaluate the impact of immune cells on senescence at both systemic and holistic levels.

## Conclusions

This study expertly evaluated the metabolically driven senescence of neutrophils in terms of ultrastructure, biological function, and intrinsic diversity. Using flow cytometry, we demonstrated at the cellular level that senescent neutrophils exhibited decreased chemotaxis, phagocytosis, ROS release, and NET formation. Age-related heterophenotypes appeared at the level of cell surface markers, which may serve as an entry point to identify and evaluate neutrophil senescence status.

Concurrently, we further investigated the potential mechanism of ceramide intervention in neutrophil senescence, verifying the expression levels of RNA, protein, and inflammatory factors. Our results indicate that ceramide plays a dual role in promoting apoptosis of senescent neutrophils and inhibiting the inflammatory cGAS-STING signaling pathway. Additionally, at the cellular ultrastructure level, we elucidated the feasibility and primary mechanism of ceramide intervention in neutrophil senescence: affecting mitochondrial integrity.

Such explorations potentially pave the way for novel perspectives in evaluating and intervening in immune senescence, and ultimately, organismal aging.

**Figure 8:**
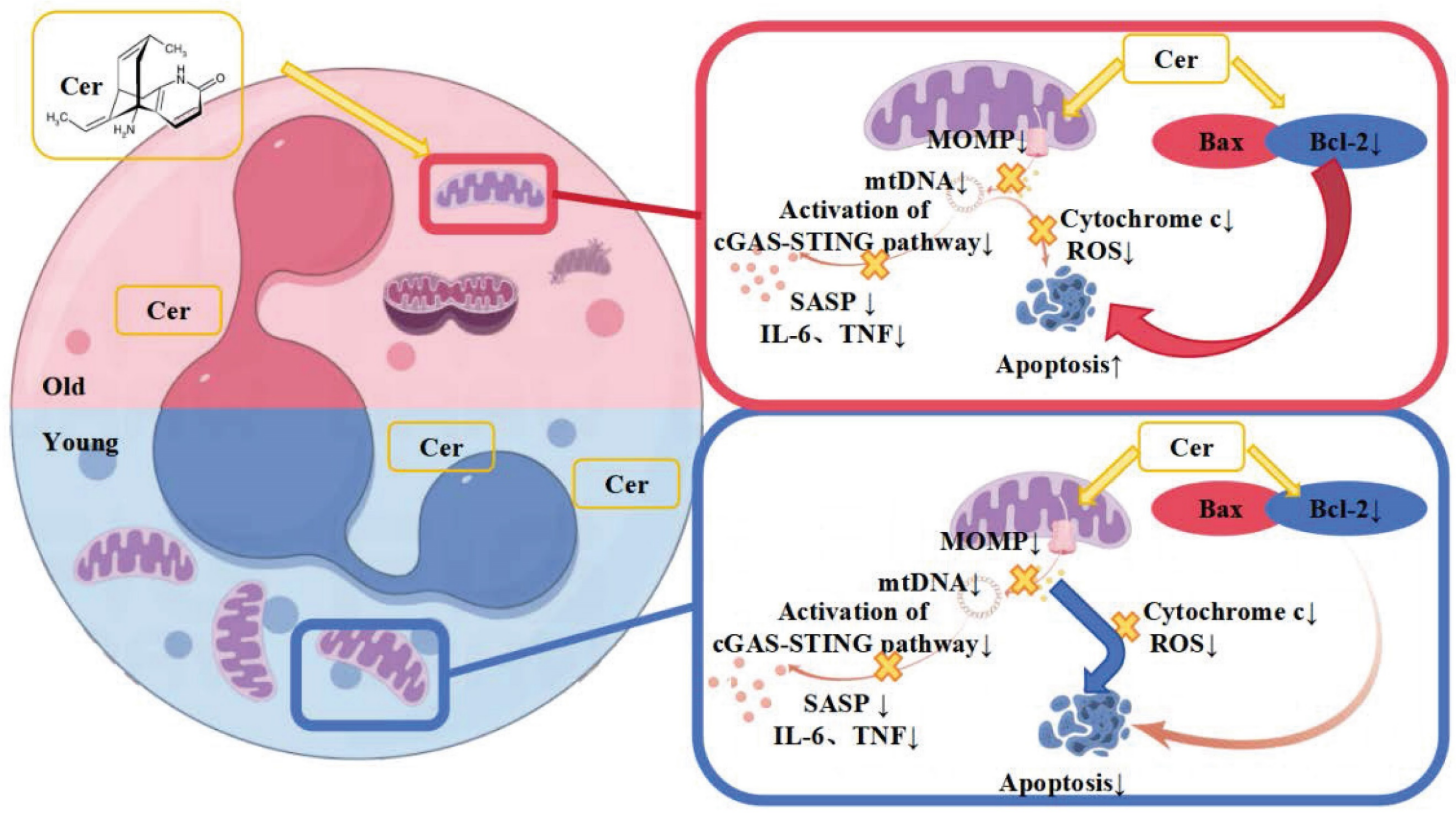
Proposed differential mechanisms by which ceramide mixture regulates apoptosis in young and aged neutrophils.

Therefore, based on the above phenomena and experimental results, we hypothesize that the different mechanisms by which the ceramide mixture regulates apoptosis *in vitro* in young and aged PMNs lie in the differential roles of mitochondria and apoptosis-related proteins (**Figure 8**).

## Supplementary Material

Supplementary figures and video legends.

Supplementary videos.
