# Supplementary material for: Ceramide Complex Ameliorates Metabolically Driven Neutrophil Senescence by Regulating Apoptosis via the cGAS-STING Pathway
Source: Int J Med Sci. 2025 Feb 10;22(5):1124–37. doi: 10.7150/ijms.104801 (PMC11866534; doi:10.7150/ijms.104801)
Supplement: Supplementary file 1 — Supplementary figures and video legends. [file ijmsv22p1124s1.pdf]

Supporting Information for

Ceramide Complex Ameliorates Metabolically Driven Neutrophil Senescence by Regulating Apoptosis via the cGAS-STING Pathway

Xi Gao, Cheng Lu, Kaixuan Wan, Chunfang Zheng, Linbin Li, Xin Zhang, Bingwei Sun*

*** Corresponding author**

Email: sunbinwe@hotmail.com

This file includes:

Figures S1 to S4

Video S1 to S6

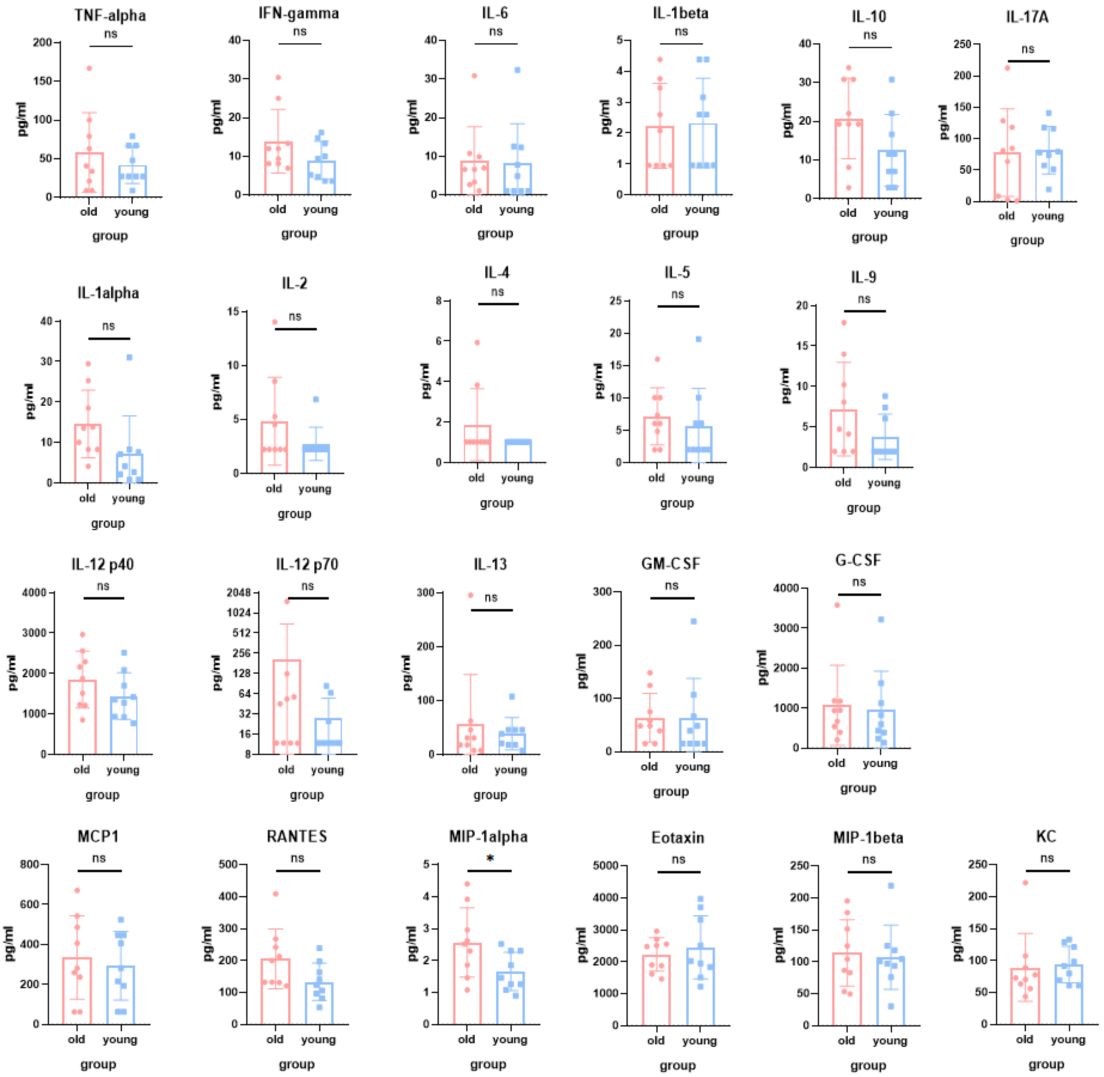

Fig. S1 Levels of plasma inflammatory factors in mice. Results of Luminex multifactor assay showing the concentrations of plasma inflammatory factors in mice(n=9).

A

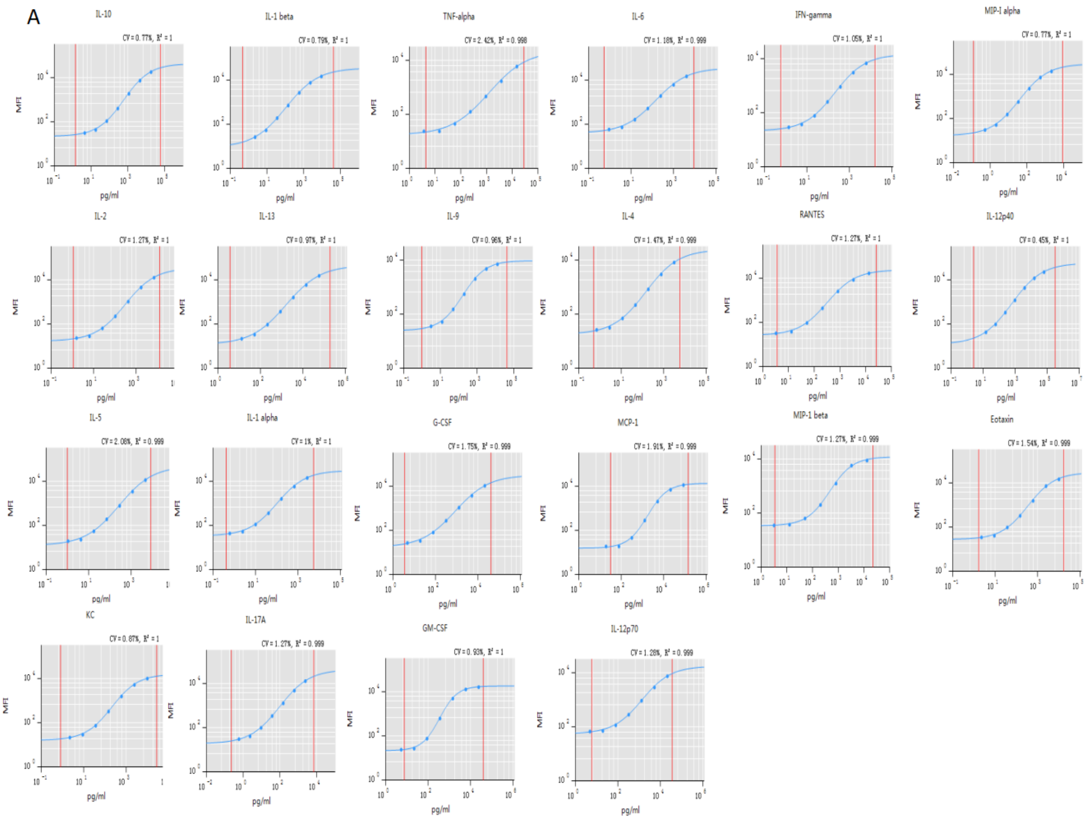

B

cytokine	lower and upper limit of detection
IL-1beta	DC=(0. 48, 40941)
TNF-alpha	DC=(4. 45, 28369)
IL-9	DC=(0. 98, 40997)
IFN-gamma	DC=(0. 59, 15882)
IL-2	DC=(1. 11, 12902)
IL-13	DC=(3. 75, 185996)
IL-6	DC=(0. 54, 9182)
IL-4	DC=(0. 51, 5852)
MCP-1	DC=(31. 61, 141106)
IL-5	DC=(1. 00, 8281)
IL-1 alpha	DC=(0. 41, 5456)
G-CSF	DC=(3. 51, 40889)
RANTES	DC=(3. 62, 25760)
IL-10	DC=(1. 42, 59494)
KC	DC=(0. 82, 27055)
IL-17A	DC=(0. 22, 6957)
GM-CSF	DC=(7. 56, 39557)
Eotaxin	DC=(1. 68, 16986)
MIP1-beta	DC=(3. 45, 22052)
IL-12p40	DC=(2. 64, 321911)
MIP1-alpha	DC=(0. 12, 8786)
IL-12p70	DC=(5. 94, 35793)

Fig. S2 A supplementary explanation of the Luminex experiments (A) a mention of coefficient of correlation R of standard curves of each cytokine. (B)information regarding lower and upper limit of detection of each cytokine.

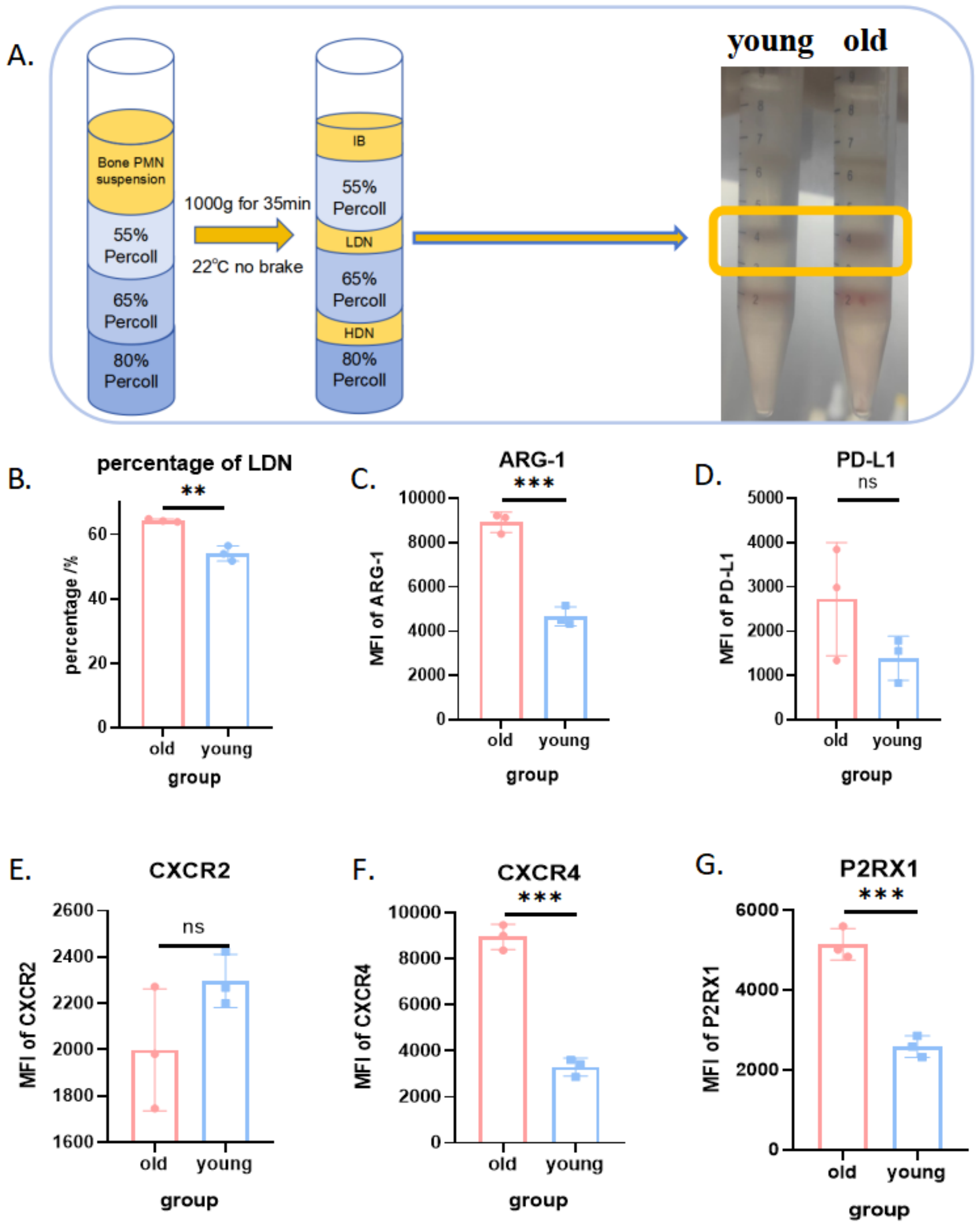

Fig. S3 Heterogeneity of neutrophils (A)Left panel: Schematic diagram of LDN isolation by Percoll density gradient method. Right panel: Separation of cell suspension obtained by Percoll density gradient method after neutrophil purification from aged and young mice. (B) Quantification of LDN percentage based on total LDN and HDN counts using a cell counter (n=3). (C) Flow cytometric analysis of ARG-1 expression on the neutrophil cell membrane surface in old and young mice (n=3). (D) Flow cytometric analysis of PD-L1 expression on the neutrophil cell membrane surface in old and young mice (n=3). (E-G) Flow cytometric analysis of CXCR2, CXCR4, and P2RX1 expression on the neutrophil cell membrane surface in old and young mice (n=3 for each).

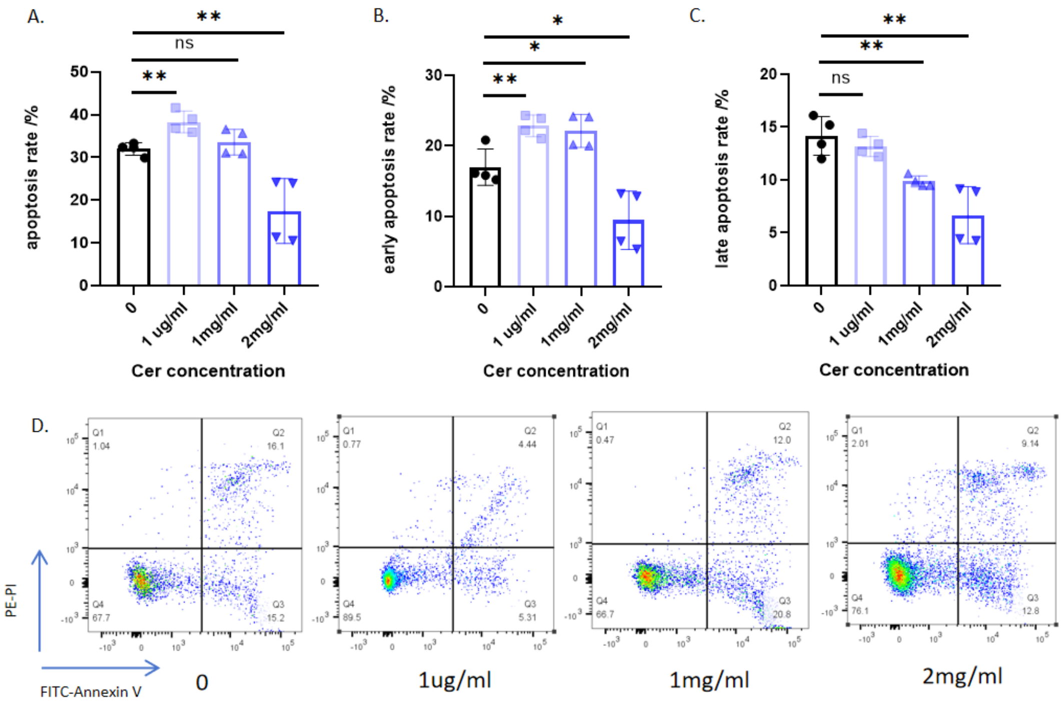

Fig. S4 Different concentrations of ceramide mixtures intervened in vitro apoptosis of neutrophils in young mice. (A)Flow cytometry showed apoptosis level of neutrophils of young mice cultured with different concentrations of ceramide mixtures in vitro for 4h(n=4). (B)Flow cytometry showed early apoptosis level of neutrophils of young mice cultured with different concentrations of ceramide mixtures in vitro for 4h(n=4). (C)Flow cytometry showed late apoptosis level of neutrophils of young mice cultured with different concentrations of ceramide mixtures in vitro for 4h(n=4). (D)Representative flow cytometry scatter plot showed the expression of apoptosis-related Annexin V-PI in neutrophils of young mice after 4 hours of culture with different concentrations of ceramide mixtures in vitro.

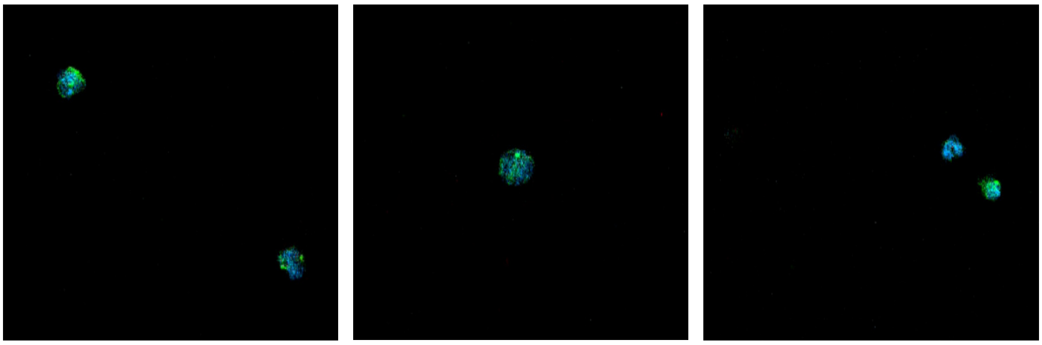

Supplemental video S1-3. 3D scanning of neutrophils in aged mice. Immunofluorescence confocal imaging showing the intracellular structure of neutrophils in aged mice: green (cell membrane), blue (nucleus), red (mitochondria). Zeiss LSM 900 confocal microscope was used to observe the blood flow. Scale bar, 10um.

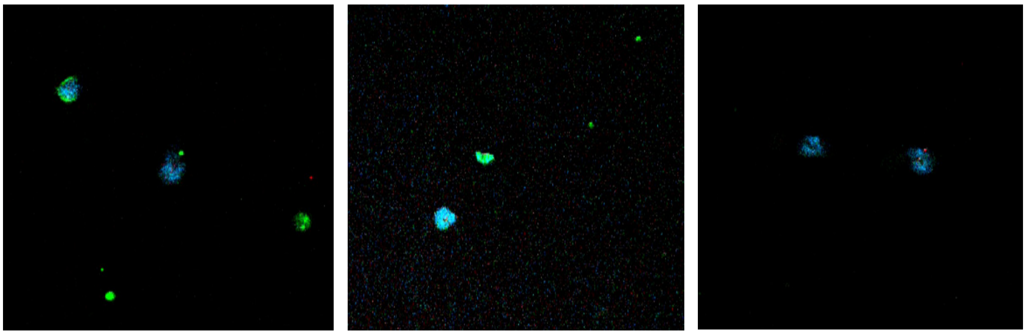

Supplemental video S4-6. 3D scanning of neutrophils in young mice. Immunofluorescence confocal imaging showing the intracellular structure of neutrophils in young mice: green (cell membrane), blue (nucleus), red (mitochondria). Zeiss LSM 900 confocal microscope was used to observe the blood flow. Scale bar, 10um.
